# Retrospective estimation of the time-varying effective reproduction number for a COVID-19 outbreak in Shenyang, China: An observational study

**DOI:** 10.1097/MD.0000000000038373

**Published:** 2024-05-31

**Authors:** Peng Li, Lihai Wen, Baijun Sun, Wei Sun, Huijie Chen

**Affiliations:** a Department of Infectious Disease, Shenyang Municipal Center for Disease Control and Prevention, Shenyang, Liaoning Province, China; b Department of National Health, China Medical University, Shenyang, Liaoning Province, China.

**Keywords:** effective reproduction number, generation time, SARS-CoV-2 Omicron variant of concern, series interval

## Abstract

The time-varying effective reproduction number *R*_*e*_(*t*) is essential for designing and adjusting public health responses. Retrospective analysis of *R*_*e*_(*t*) helps to evaluate health emergency capabilities. We conducted this study to estimate the *R*_*e*_(*t*) of the Corona Virus Disease 2019 (COVID-19) outbreak caused by SARS-CoV-2 Omicron in Shenyang, China. Data on the daily incidence of this Corona Virus Disease 2019 outbreak between March 5, 2022, and April 25, 2022, in Shenyang, China, were downloaded from the Nationwide Notifiable Infectious Diseases Reporting Information System. Infector–infectee pairs were identified through epidemiological investigation. *R*_*e*_(*t*) was estimated by R-studio Package “EpiEstim” based on Bayesian framework through parameter and nonparametric method, respectively. About 1134 infections were found in this outbreak, with 20 confirmed cases and 1124 asymptomatic infections. Fifty-four infector–infectee pairs were identified and formed a serial interval list, and 15 infector–infectee pairs were included in the generation time table. *R*_*e*_(*t*) calculated by parameter and nonparametric method all peaked on March 17, 2022, with a value of 2.58 and 2.54 and decreased to <1 after March 28, 2022. There was no statistical difference in the *R*_*e*_(*t*) distribution calculated using the 2 methods (*t* = 0.001, *P* > .05). The present study indicated that the decisive response of Shenyang, China, played a significant role in preventing the spread of the epidemic, and the retrospective analysis provided novel insights into the outbreak response to future public health emergencies.

## 1. Introduction

The basic reproduction number (R0) is a well-known epidemiological concept that quantifies the spread of infectious diseases. It is defined as the mean number of secondary infections seeded in a primary case.^[1]^ The transmissibility of an infection can be quantified based on its R0 value in a completely susceptible host population where no one is either immune or vaccinated.^[2]^ However, as intervention measures are put in place and a certain proportion of the population gains immunity, transmissibility is better quantified by the time-varying effective reproduction number-*R*_*e*_(*t*), which is a time-dependent quantity that accounts for the population’s reduced susceptibility owing to the natural history of an infection and the use of vaccines.^[3]^

*R*_*e*_(*t*) is essential for designing and adjusting public health responses.^[4]^ Previous studies have evaluated the *R*_*e*_(*t*) of Corona Virus Disease 2019 (COVID-19), but *R*_*e*_(*t*) was greatly affected by local prevention and control measures.^[4–10]^ Between March 5, 2022, and April 25, 2022, a COVID-19 outbreak caused by SARS-CoV-2 Omicron (as confirmed by gene sequencing) occurred in Shenyang, China. However, to date, there have been no reports of the *R*_*e*_(*t*) of COVID-19 in Shenyang, China. Retrospective analysis can not only evaluate *R*_*e*_(*t*), but also help evaluate health emergency capabilities. The “EpiEstim” software package developed by the Cori A team at Imperial College in the UK^[11]^ is based on the Bayesian framework method and can be used to estimate the *R*_*e*_(*t*) during epidemics, this method has been currently widely used.^[12,13]^ Generation time (GT) and serial interval (SI) are important parameters for calculating *R*_*e*_(*t*). Therefore, in this study, we estimated the *R*_*e*_(*t*) of the outbreak that occurred in Shenyang based on the “EpiEstim” software package using SI and GT corresponding to the parameter and nonparametric methods, and evaluated the actual effectiveness of the prevention and control of the COVID-19 outbreak in Shenyang.

## 2. Materials and methods

### 2.1 . Data

Incidence data for this COVID-19 outbreak in Shenyang, China between March 5, 2022, and April 25, 2022, were extracted from the Nationwide Notifiable Infectious Diseases Reporting Information System (NIDRIS). The data were used under a license and were not publicly available. Infector–infectee pairs were identified through epidemiological investigations. An infector–infectee pair consists of an infector and an infected infectee. The infectee in the infector–infectee pair was in close contact with the infector, and had no history of contact with other infected persons. The study protocol was approved by the Ethics Committees of Shenyang Municipal Center for Disease Control and Prevention (No. 202301002).

### 2.2. SI list of infector–infectee pairs

SI is defined as the time between the onset of the primary (infector) and secondary (infectee).^[14]^ In the present study, the onset of symptoms in asymptomatic individuals referred to the moment when the SARS-COV-2 reverse transcription-polymerase chain reaction (RT-PCR) test yielded a positive result. Asymptomatic persons carry the SARS-COV-2 virus but do not show any symptoms (e.g., fever or gastrointestinal or respiratory symptoms) or significant abnormalities on chest radiography.^[15]^ An SI list was created for each infector–infectee pair.

### 2.3. GT table of infector–infectee pairs

GT is defined as the interval between the moment when one person is infected (infector) and the moment someone else is infected (infectee).^[16]^ In other words, it is the time between infection events in an infector–infectee pair. The GT table includes 5 variables: EL, ER, SL, SR, and type. EL and ER indicate the lower and upper bounds of the dates of exposure in the infectors, respectively; SL and SR indicate the lower and upper bounds of the dates of exposure in the infected individuals, respectively. The type column contains entries 0, 1, and 2, which correspond to double interval-censored, single interval-censored, and exact observations, respectively.

### 2.4. *Calculation of R*_*e*_*(t*)

As assumed by Cori et al, once infected, individuals have an infectivity profile given by a probability distribution ω_τ_, which typically depends on individual biological factors such as pathogen shedding and symptom severity.^[11]^ *R*_*e*_(*t*) can be estimated based on the ratio of the number of new infectors generated at time t to the total number of infected individuals at time τ, weighted by the infectivity function ω_τ_. *R*_*e*_(*t*) is the average number of secondary cases that each infected individual would infect if the conditions remained the same as they were at time *t*.

Thus, *R*_*e*_(*t*) can be defined as follows:


$$\begin{array}{l} R\left(t\right)=I\left(t\right){\left[\underset{0}{\overset{t}{\int }}\;I\left(t-\tau \right)\omega \left(\tau \right)d\tau \right]}^{-1}. \end{array}$$

The infectivity profile ω_τ_ is equivalent to a GT distribution. Then probabilistic estimates of *R*_*e*_(t) can be resolved in a Bayesian framework as implemented in the “EpiEstim” package.^[11]^

The parametric and nonparametric methods corresponding to “parametric_si” and “si_from_sample” included in the “estimate_R” function were used to calculate *R*_*e*_(*t*), respectively. *R*_*e*_(*t*) was calculated for weekly sliding windows. When using the parametric method, the “est.GT” function in the “R0” package was used to convert the SI list into a GT distribution, marked as GT^a^ distribution. The “estimate_R” function with the “parametric_si” method in the “EpiEstim” package was used to calculate the *R*_*e*_(*t*). When using the nonparametric method, the “dic.fit.mcmc” function in the “coarseDataTools” was used to resample the GT table of each infector–infectee pair to obtain the available GT distribution, marked as GT^b^ distribution. The “estimate_R” function with the “si_from_sample” method in the “EpiEstim” package was used to calculate the *R*_*e*_(*t*). We did not stratify incidence by “imported” versus “local” transmission events, but the relative influence of different GT distribution on *R*_*e*_(*t*) should not be affected by this lack of stratification.^[17]^ The calculation process is presented in the Supplementary file 1, Supplemental Digital Content, http://links.lww.com/MD/M675.

### 2.5. Statistical analysis

The R-Studio 2022.02.3 + 492 software was used to calculate *R*_*e*_(*t*). SPSS software (version 23.0) was used for the statistical analysis. The Student *t* test was used for comparisons between the 2 groups. The significance level was *P* < .05.

## 3. Results

**Figure 1.**
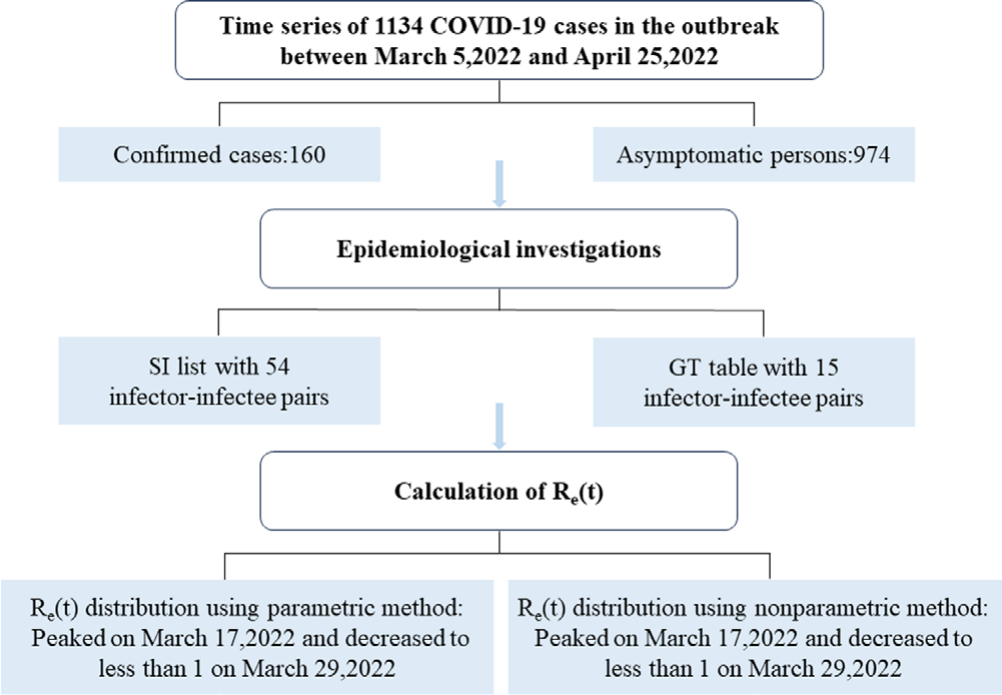
Study flowchart showing the main process of *R*_*e*_(*t*) estimation. First, an epidemiological investigation was conducted on 1134 cases that reported in this outbreak, and 54 infector–infectee pairs were extracted in the SI list and 15 infector–infectee pairs were extracted in the GT table. Then, the parametric and nonparametric method were used to calculate the *R*_*e*_(*t*), respectively. GT = generation time, SI = series interval.

The study flowchart is illustrated in Figure 1.

### 3.1. Time series

**Figure 2.**
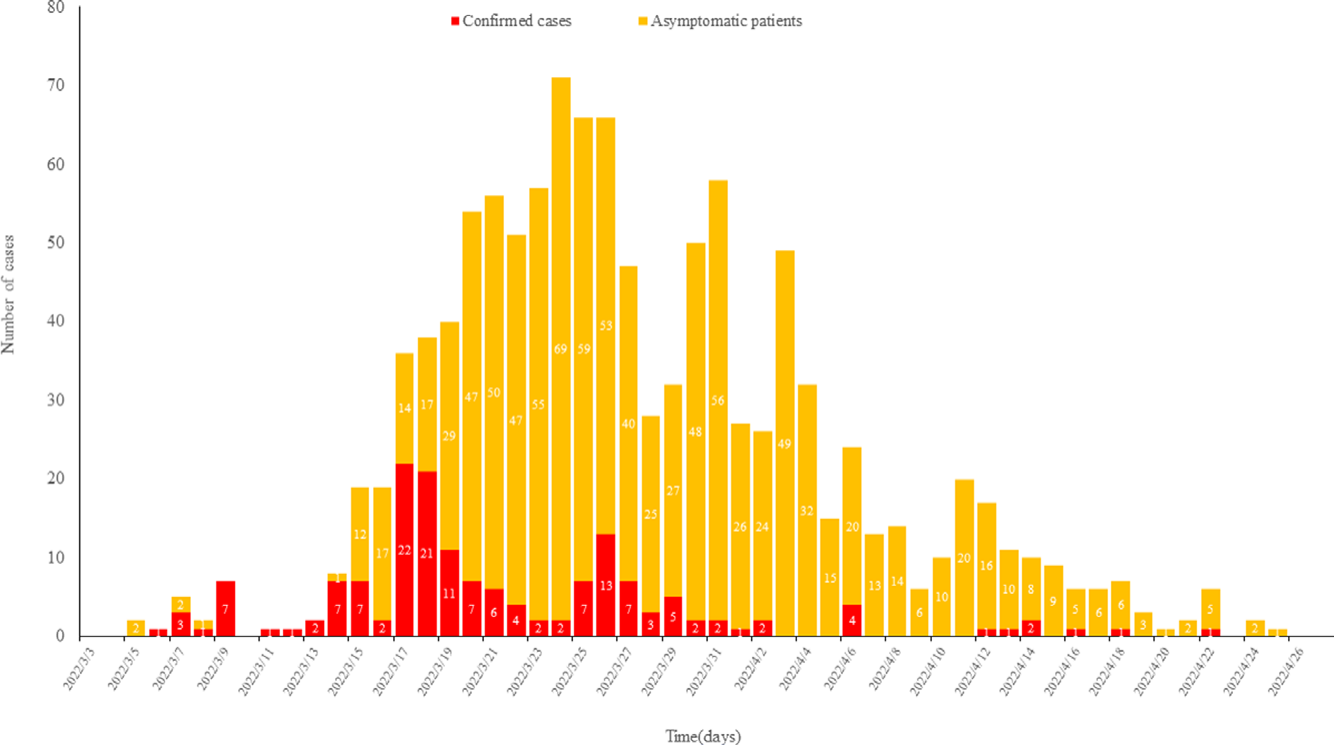
Time series of COVID-19 from March 5, 2022 to April 25, 2022 in Shenyang. Red pillar represented the number of newly confirmed cases per day. Orange pillar represented the number of newly asymptomatic persons per day.

A total of 1134 SARS-CoV-2 infectors were reported in Shenyang between March 5, 2022, and April 25, 2022, including 160 confirmed cases and 974 asymptomatic persons. The number of cases increased in the waves since the detection of 2 infected persons on March 5, 2022, and peaked on March 24, 2022, and then decreased in waves. The last case was detected on April 25, 2022, see Figure 2.

### 3.2. SI list and GT table of infector–infectee pairs

According to the identification criteria for infector–infectee pairs, 54 pairs were identified during the initial stage of the outbreak. The SI list was made based on the onset times of the 54 infector–infectee pairs (Table 1).

**Table 1:** SI list of 54 infector–infectee pairs from the COVID-19 outbreak in Shenyang, China.

Number of pair	SI	Number of pair	SI	Number of pair	SI
1	2	19	2	37	1
2	2	20	2	38	3
3	2	21	0	39	3
4	2	22	2	40	2
5	2	23	2	41	1
6	4	24	2	42	2
7	4	25	2	43	4
8	4	26	2	44	3
9	3	27	2	45	2
10	3	28	2	46	3
11	4	29	2	47	2
12	4	30	2	48	4
13	3	31	2	49	7
14	0	32	2	50	0
15	0	33	3	51	6
16	0	34	0	52	6
17	10	35	3	53	1
18	1	36	2	54	11

SI = series interval.

As it was impossible to determine the initial exposure time exactly if an infected person had been in contact with both his/her infector and his/her infectee for a long time, infector–infectee pairs with this infected person were excluded when generating the GT table. For example, if case B was the infected person mentioned above, cases A and B were colleagues who met each other every day, cases B and C were a couple who met each other every day, case A infected case B, and case B infected case C, then the infector–infectee pairs with case B were excluded. After exclusion, 15 pairs were included in the GT table (Table 2).

**Table 2:** GT table of 15 infector–infectee pairs from the COVID-19 outbreak in Shenyang, China.

Number of pair	EL	ER	SL	SR	Type
1	0	2	6	6	1
2	3	6	8	8	1
3	6	6	6	10	1
4	6	10	9	9	1
5	9	9	9	11	1
6	9	9	9	12	1
7	9	9	9	13	1
8	6	6	7	18	1
9	9	9	9	15	1
10	9	9	9	14	1
11	9	9	9	17	1
12	9	9	9	12	1
13	9	9	11	12	1
14	9	12	9	13	0
15	9	14	9	16	0

GT = generation time.

### 3.3. *The time-varying effective reproduction number R*_*e*_*(t*)

**Figure 3.**
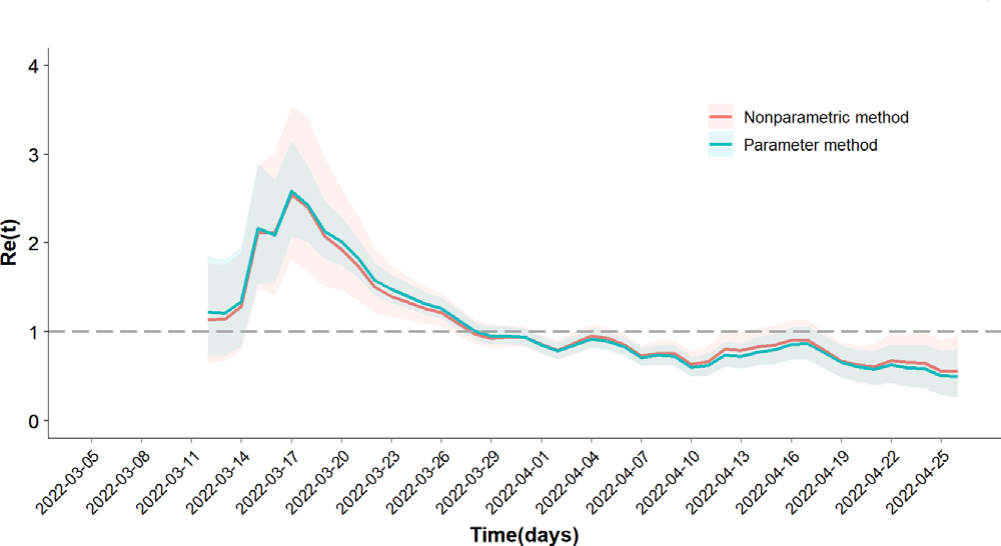
*R*_*e*_(*t*) distribution varying with time on weekly sliding windows. The blue line and red line represented the *R*_*e*_(*t*) distribution varying with time on weekly sliding windows calculated using the parametric and nonparametric method, respectively. It can be seen that the *R*_*e*_(*t*) distributions varying with time are approximated by the 2 methods.

As shown in Figure 3, *R*_*e*_(*t*) varied with time. *R*_*e*_(*t*) calculated by either the parametric method or the nonparametric method peaked on March 17, 2022, with values of 2.58 and 2.54, respectively. *R*_*e*_(*t*) calculated using the parametric method decreased to <1 on March 29, 2022, and then remained <1. *R*_*e*_(*t*) calculated using the nonparametric method decreased to <1 on March 28, 2022, and then remained <1. There was no statistical difference between the *R*_*e*_(*t*) distributions calculated using the 2 methods (*t* = 0.001, *P* > .05). The average *R*_*e*_(*t*) value as determined by the parametric method was 1.08, with a range from 0.50 to 2.58 (median: 1.07; IQR: 1.00–1.15) and the average *R*_*e*_(*t*) value as determined by the nonparametric method was 1.08, with a range from 0.58 to 2.55 (median: 1.07; IQR: 0.97–1.17).

## 4. Discussion

Previous studies have indicated that viral loads in asymptomatic persons and symptomatic SARS-CoV-2 patients may be similar^[17–21]^ and that asymptomatic persons are infectious but might be less infectious than confirmed cases.^[22]^ In view of the fact that asymptomatic persons are a potential source of substantial spread within the community setting^[23]^ and that more than 85% of cases were asymptomatic in the Shenyang outbreak, in the present study, both confirmed cases and asymptomatic persons were listed as infectors for the evaluation of the infectivity of SARS-CoV-2.

Liu and Rocklov^[4]^ found that the average *R*_*e*_(*t*) for COVID-19 was 3.4, ranging from 0.88 to 9.4 (median: 2.8; IQR: 2.03–3.85). The present study revealed that the average *R*_*e*_(*t*) value was 1.08, which was significantly lower than that reported by Liu and Rocklov.^[4]^ In addition, compared with the estimation of *R*_*e*_(*t*) in other provinces and cities in China by You et al^[7]^ the value determined in the present study was also significantly lower. This finding may be related to policies to prevent importation and internal outbreaks in China and strict control measures for isolating infectors and cutting off transmission routes taken by Shenyang, China.

The municipal government responded quickly during the most recent COVID-19 outbreak in Shenyang, which began on March 5, 2022. To control the source of infection, infectors were transferred to the negative-pressure isolation ward of the designated hospital for treatment until they were discharged. The close contacts of the infectors were transferred to the designated isolation hotel for isolation for at least 14 days.^[24,25]^ To cutoff the routes of transmission, from March 14, 2022, entertainment venues were closed and schools and universities switched to online lectures. From March 17, 2022, except for the normal operation of markets and enterprises that are necessary to meet basic necessities, all commerce, as well as urban public transport, including the subway, bus, and tram, was suspended. From March 23, 2022, the city was divided into lockdown, control, and prevention areas. In lockdown areas, residents did not leave the house and door-to-door services were provided. In the control areas, residents did not leave, and gathering was strictly prohibited. In prevention areas, residents followed the principle of “2 points and 1 line” (from residence to work). The *R*_*e*_(*t*) value dropped sharply after reaching the peak on March 17, 2022, thanks to these prevention and control measures and decreased to <1 after March 28, 2022. This indicated that the decisive response played a significant role in preventing the spread of the epidemic.

To identify and isolate infectors as soon as possible, multiple rounds of full-staff SARS-CoV-2 RT-PCR screening were initiated on March 17, 2022, and more infectors were successively detected. However, with the implementation of measures to control the source of infection and cutoff transmission routes, the number of new infectors per day gradually decreased after reaching a peak on March 24, 2022. The number of new infectors rebounded on March 30, 2022, March 31, 2022, and April 3, 2022, which may be related to the regional spread caused by infectors with a long incubation period. However, because *R*_*e*_(*t*) was continuously <1, such a rebound did not affect the epidemic trend. The final rounds of full-staff SARS-CoV-2 RT-PCR screening showed that the number of new infectors decreased significantly.

In this outbreak, the collaboration of multiple departments, as well as the flexible application of measures to control the source of infection, cutoff the pathogen transmission, and nucleic acid test for all staff, not only effectively controlled the spread of the epidemic, but also provided a very good demonstration of the response to future public health emergencies.

However, despite the *R*_*e*_(*t*) of this outbreak being retrospectively evaluated and subsequent analysis being conducted in this study, which provided valuable empirical support for an uncertain outbreak of infectious disease, there were certain limitations that require acknowledgments. First, during the COVID-19 outbreak, the epidemiological investigation in the context of quarantine control might be hindered by infected individuals who chose to conceal or provide false information for self-protection of shirking accountability. Despite our investigators’ diligent efforts to verify such information, it was inevitable that there would be omissions, which could impact the identification of infector–infectee pairs. Second, the number of infector–infectee pairs identified to form the SI list and GT table was small. The identification of infector–infectee pairs obtained by epidemiological investigation was challenging due to the complexity of transmission. We will persist in exploring and enhancing the evaluation methodology to alleviate these biases.

## 5. Conclusions

The present study indicated that the decisive response of Shenyang, China, played a significant role in preventing the spread of the epidemic, and the retrospective analysis provided novel insights into the outbreak response to future public health emergencies.

## Acknowledgments

We would like to thank the frontline epidemiological investigator and medical staff, the technicians in the microbiology laboratory department of the Shenyang Center for Disease Control and Prevention, and the patients for providing epidemiological information for this research.

## Author contributions

**Conceptualization:** Peng Li, Baijun Sun, Wei Sun, Huijie Chen.

**Data curation:** Peng Li, Lihai Wen.

**Methodology:** Peng Li, Baijun Sun, Wei Sun, Huijie Chen.

**Resources:** Peng Li, Lihai Wen, Baijun Sun.

**Software:** Peng Li, Wei Sun, Huijie Chen.

**Writing – original draft:** Peng Li, Huijie Chen.

**Formal analysis:** Lihai Wen, Huijie Chen.

**Investigation:** Lihai Wen, Baijun Sun, Huijie Chen.

**Supervision:** Wei Sun, Huijie Chen.

**Writing – review & editing:** Wei Sun, Huijie Chen.

## Supplementary Material


