# Supplementary material for: Retrospective estimation of the time-varying effective reproduction number for a COVID-19 outbreak in Shenyang, China: An observational study
Source: Medicine (Baltimore). 2024 May 31;103(22):e38373. doi: 10.1097/MD.0000000000038373 (PMC11142808; doi:10.1097/MD.0000000000038373)
Supplement: Supplementary file 1 [file medi-103-e38373-s001.docx]

**The calculation process of R_e_(t) in RStudio**

**Data preparation：**

TS.xlsx contains incidence data. SI.xlsx is the SI list mentioned in the manuscript. GT .xlsx is the GT table mentioned in the manuscript.

# install.packages, as follows:

#install.packages("R0")

#install.packages("openxlsx")

#install.packages("ggplot2")

#install.packages("EpiEstim")

#install.packages("coarseDataTools")

#install.packages("fitdistrplus")

**Calculate R_e_(t) by parameter method**

#Convert SI into generation distribution, as follows:

SI_data <-read.xlsx("SI.xlsx",sheet = 1)

GT1<-est.GT(serial.interval=SI_data$SI)

#Fitting:

Test fitting distribution was LogNorm distribution with mean = 2.84 and sd = 2.22, as follows:

f.a<-fitdist(GT1$GT,distr = "lnorm",method = "mle")

f.b<-fitdist(GT1$GT,distr = "norm",method = "mme")

f.c<-fitdist(GT1$GT,distr = "gamma",method = "mme")

f.d<-fitdist(GT1$GT,distr = "exp",method = "mme")

par(mfrow=c(2,2))

fp=list(f.a,f.b,f.c,f.d)

denscomp(fp,addlegend = T,xlegend = "bottomright")

cdfcomp(fp,addlegend = T,xlegend = "bottomright")

qqcomp(fp,addlegend = T,xlegend = "bottomright")

ppcomp(fp,addlegend = T,xlegend = "bottomright")

sy <- read.xlsx("TS.xlsx",sheet = 1)

sy$date <- as.Date(sy$date,origin = "1899-12-30")

#Calculate R_e_(t) , as follows:

Rt1 <- estimate_R(incid = sy$case,

method = "parametric_si",

config = make_config(list(

mean_si = 2.84, std_si = 2.22)))

#Extract R-means and 95% confidence intervals, as follows:

eRt <- Rt1$R$`Mean(R)`

Rt1_tiqu <- data.frame(R=Rt1$R$`Mean(R)`,

RL=Rt1$R$`Quantile.0.025(R)`,

RH=Rt1$R$`Quantile.0.975(R)`,

date=seq.Date(from=as.Date(sy$date[nrow(sy)])-length(eRt)+1,

to = as.Date(sy$date[nrow(sy)]),by="day"))

# R_e_(t) of the first 7 days were filled with Na, as follows:

Rt1_bind <- rbind(Rt1_tiqu,

data.frame(R=rep(NA,7),RL=rep(NA,7),RH=rep(NA,7), date=seq.Date(as.Date("2022-03-05"),as.Date("2022-03-11"),"day")))

**Calculate R_e_(t) by non-parameter method**

# Build data-frame of GT_ data, as follows:

EL<-c(0,3,6,6,9,9,9,6,9,9,9,9,9,9,9)

ER<-c(2,6,6,10,9,9,9,6,9,9,9,9,9,12,14)

SL<-c(6,8,6,9,9,9,9,7,9,9,9,9,11,9,9)

SR<-c(6,8,10,9,11,12,13,18,15,14,17,12,12,13,16)

type<-c(1,1,1,1,1,1,1,1,1,1,1,1,1,0,0)

GT_data<-data.frame(EL,ER,SL,SR,type)

# Resampling by MCMC, as follows:

MCMC_seed <- 1

GT.fit <- dic.fit.mcmc(dat = GT_data, dist = "G",

init.pars = init_mcmc_params(GT_data, "G"),burnin = 1000,

n.samples = 5000, seed = MCMC_seed)

#Coarse2estim function convert, as follows:

GT2 <- coarse2estim(GT.fit, thin = 10)$si_sample

#Calculate R_e_(t) , as follows:

overall_seed <- 2

Rt2 <- estimate_R(incid=sy$case,

method = "si_from_sample",

si_sample = GT2,

config = make_config(list(n2 = 100,

seed = overall_seed)))

#Extract R-means and 95% confidence intervals, as follows:

eRt <- Rt2$R$`Mean(R)`

Rt2_tiqu <- data.frame(R=Rt2$R$`Mean(R)`,

RL=Rt2$R$`Quantile.0.025(R)`,

RH=Rt2$R$`Quantile.0.975(R)`,

date=seq.Date(from=as.Date(sy$date[nrow(sy)])-length(eRt)+1,

to = as.Date(sy$date[nrow(sy)]),by="day"))

# R_e_(t) of the first 7 days were filled with Na, as follows:

Rt2_bind <- rbind(Rt2_tiqu,

data.frame(R=rep(NA,7),RL=rep(NA,7),RH=rep(NA,7),

date=seq.Date(as.Date("2022-03-05"),as.Date("2022-03-11"),"day")))

# R_e_(t) curves were plotted by ggplot2, as follows:

Rt1_bind<-transform(Rt1_bind,Group="Parameter method")

Rt2_bind<-transform(Rt2_bind,Group="Nonparametric method ")

Rt<-rbind(Rt1_bind,Rt2_bind)

colnames(Rt)[5] <- "method"

p <- ggplot(Rt,aes(x=date,y=R,group=method,color=method))+

geom_line(size=1,alpha=1)+

geom_ribbon(aes(ymin=RL,ymax=RH,fill=method),alpha=0.1,linetype = 1,color=NA) +

labs(title="",x="Time(days)",y="Re(t)")+

theme(legend.position = c(0.8,0.8),

axis.title = element_text(size=13,face="bold",colour = "black",vjust = 0),

axis.line = element_line(size=0.2, colour = "black"),

panel.background = element_rect(fill="transparent"),

rect=element_blank(),

panel.border = element_blank(),

panel.grid.major = element_blank(),

panel.grid.minor = element_blank(),

axis.text.x = element_text(size=10,angle=45,hjust = 1,colour = "black",

margin=unit(c(0.1,0.1,0.1,0.1),"cm")),

axis.text.y = element_text(size=12,colour = "black",

margin=unit(c(0.1,0.1,0.1,0.1),"cm")),

title = element_text(size=10,colour = "black"),

strip.text = element_text(size=10),

plot.title = element_text(hjust = 0.5,size=10,colour="black"),

legend.title = element_blank(),

legend.text = element_text(size=10,color="black"),

axis.ticks.x = element_line(size = 0.1,lineend = c(2,2,2)))+

scale_x_date(breaks = seq(as.Date("2022-03-05"), as.Date("2022-04-26"), by="3 day")) +

coord_cartesian(ylim=c(0,4))+

scale_y_continuous(breaks=round(seq(0,4, by =0.5),0))+

geom_hline(aes(yintercept=1),linetype="longdash",size=0.8,color="darkgrey")

p
